# Book Review

**Published:** 1930

**Authors:** 


### Reviews of Books

**On Faith and Science in Surgery.** By Sir John Bland-Sutton, Bart. Pp. xii., 109. 40 illustrations. London: William Heinemann Ltd. 1930. Price 7s. 6d.—This small volume consists of ten addresses delivered between 1925 and 1930 and put together under the rather misleading title above quoted. Curiosities of Science distantly related to Surgery would be perhaps a more accurate description of its contents. When a surgeon has become notorious as an interesting and witty speaker, he must find it difficult to choose fresh subjects for his addresses and to name them with attractive titles. Bland-Sutton more than any living man has inherited John Hunter's keen and insatiable curiosity about all the inexplicable phenomena of animal life, and therefore he takes some curious phase of normal or abnormal biology as his theme. The address on ligatures refers to the sewing of tailor birds and to the use of ants' mandibles as skin clips. Two addresses on the faith in drugs and physic are illuminated by references to the ancient practice of physic and to the natural concretions of animals. These latter have always had great vogue as healing drugs. The chapter on twins and monsters and that on the psychology of animals swallowed alive are full of absorbing interest. We are constantly reminded throughout the book of the marvellous versatility of the author. To begin with he is a zoologist whose resting thoughts always seem to hark back to the denizens of the deep sea or the wonders of the Zoo. Then he is an inquiring anatomist who has never ceased to ask questions and to find answers about such problems as those presented by hydrocephalus. He has been a pioneer in surgery; he tells the tale of how gall-bladders first began to be taken out; and a gynæcologist who unravelled the mystery of tubal moles and did so much for the perfecting of hysterectomy. He has a great fondness for versification and these literary efforts have neither rhyme nor rhythm: also a wonderful knowledge of the Scriptures, which he quotes and refers to on all occasions and often in a manner quite at variance with orthodox interpretation.

**A Short History of the Army Medical Corps.** By COLONEL F. SMITH, C.B., C.M.G., D.S.O., R.A.M.C. Pp. 111. Illustrated. Aldershot: Gale and Polden. Price 2s. 6d.—This little volume, we are told, is written for young soldiers of the Corps who have to pass examinations in the subject. For the general reader the chief interest in the book is the fascinating account of many of the chief deeds of valour by officers and men from the Crimea down to the present day. This forms what is perhaps the most astonishing chronicle of heroic actions by men of a non-combatant corps ever put forth. Though these graphic reports take up the greater part of the book, they are only a fraction of the glorious deeds by men of the corps for which twenty-eight V.C.'s and thousands of other honours were awarded, and they form one of the best answers to the recent depreciating of war books. The author traces the gradual evolution of the Corps from the days of the old chaotic regimental system and the Crimean breakdown to the creation of the present body in 1898 near the end of the long series of Egyptian expeditions. The Corps were barely formed when we were involved in the great South African War where 22,000 men died and 76,000 were invalided. In the last Great War the Corps dealt with near nine millions of sick and wounded, and healed and sent back to the firing-line in France alone over one and a half million of wounded men, while nearly 7,000 of its officers and men gave their lives. In a subsequent edition the first chapters on the original formation of the Corps might with advantage be revised and condensed.

**Diagnosis and Treatment of Heart Disease.** By E. M. BROCKBANK, M.D., F.R.C.P. Sixth Edition. Pp. xvi., 240. Illustrated. London: H. K. Lewis & Co. Ltd. 1930. Price 7s. 6d.—The sixth edition of Dr. Brockbank's book on the *Diagnosis and Treatment of Heart Disease* is somewhat larger than the previous edition, but is still easy to read. The book should be of great use to students before sitting for their examinations. Dr. Brockbank's theory of the causation of the crescendo murmur of mitral stenosis is not accepted by cardiologists, but we notice that this fact is mentioned in the text and should therefore not be a danger to the reader. The author has taken the trouble to explain the meaning of the physical signs in simple language; a most useful thing for students who are rather apt to take things for granted and not to ask the reason why. It is a book which should be on the shelves of all the University libraries.

**The Action of Muscles.** By SIR COLIN MACKENZIE, M.D., F.R.C.S., F.R.S. Second Edition. Pp. xvi., 288. Illustrated. London: H. K. Lewis & Co. Ltd. Price 12s. 6d.—It is doubtful if this book will ever be widely read, which is a pity. It is full of interesting items of information which provoke one to think and to reconsider theories and practices which have been taken too much for granted. The excellent matter which it contains is very badly presented to the reader. Items of anatomy, pathology, physiology, comparative anatomy and treatment follow one another without any method. A drastic rearrangement of the whole volume would be well worth while. It is well printed, illustrated and indexed and will repay a patient study.

**General Practice.** By ERNEST WARD, M.D., F.R.C.S. Pp. viii., 108. London: John Bale, Sons and Danielsson, Ltd. 1930. Price 3s. 6d.—It may be an oversight, but the reviewer has failed to find in this book any statement to the effect that general practice is doomed. This alone is enough to show that the author is no ordinary man. The book is a kind of sequel to *Medical Adventure*, reviewed in these columns not long ago, and is as delightful. It is brief but full of wisdom. Take, for example, this paragraph: "Let me say again that there are three kinds of treatment. First, *physical* treatment; by rest in bed, warmth, local applications, and by drugs. Second, *mental* treatment; by no means confined to Christian Scientists. . . . Third, *social* treatment; it is necessary to create a suitable atmosphere around the patient." Happy is the young practitioner who is taught these lessons by example. If, however, this is not available, he may well be grateful to Dr. Ward for his precepts. Discerning relatives should certainly give him a copy of the book on his next birthday.

**Clinical Examination of the Nervous System.** By G. H. MONRAD-KROHN, M.D., F.R.C.P. Fifth Edition. Pp. xvi., 222. Illustrated. London: H. K. Lewis & Co. Ltd. 1930. Price 7s. 6d.—This book has been reviewed several times previously in our columns, when it was described as a most valuable guide for the examination of the nervous system. That it has now reached its fifth edition in ten years shows that its value has been appreciated and that it filled a very definite need. The present edition has again been revised and short descriptions of ventriculography and encephalography have been added.

**Visceroptosis and Allied Abdominal Conditions.** By H. BEDINGFIELD, M.D., M.R.C.P. Pp. 176. London: Oxford University Press. 1930. Price 10s. 6d.—This monograph deals with a difficult subject of the highest clinical importance, in an entirely admirable manner. It deserves the careful consideration of every general practitioner and no operating surgeon can read it without qualms, when he looks back on some of the failures of his surgical career. The book opens with a dramatic and wonderfully vivid clinical picture of the chronic abdominal invalid. The author passes in review Glénard's theories of visceroptosis and the theories which have held the field subsequent to the introduction of X-rays. These theories, in the main mechanical, are subjected to a searching and destructive criticism. The author shows that considerable range of movement and variation in position of the hollow viscera are consistent with perfect health, and that such variation is due to physiological or physical causes. In the abdominal invalid the defect is due to malfunctioning of the neuro-muscular mechanism, behind which there lies a strong constitutional factor. The autointoxication theory associated with constipation as presented by Lane is attacked and controverted. These unfortunate abdominal invalids are over and over again the victims of injudicious surgery: not infrequently a single patient has a history of multiple laparotomies to his credit and the surgeon's shame. The views of the author on surgical intervention are summarized in the following passage: "Nothing is more disastrous than surgical intervention in the chronic intestinal invalid. As a method of treatment it is useless; as a method of diagnosis it is criminal." The chapter on treatment is careful and most helpful. The book contains a very complete bibliography of the subject.

**Diathermy, Medical and Surgical, in Oto-Laryngology.** By DAN MCKENZIE, M.D., F.R.C.S.E. Pp. xiv., 184. 23 illustrations. London: Kegan Paul, Trench & Trubner. 1930. Price 10s. 6d.—This exceedingly interesting and valuable contribution to the therapeutic application of diathermy is the work of one who has long been known as an enthusiast. Nevertheless, it is essentially practical. For example, of diathermy dissection of the tonsil for malignant disease the author observes that this "is not as easy as it sounds,"—the utterance of experience so invaluable to the novice. Detailed consideration is given to the various problems that present themselves, and the views put forward are those always of the critic rather than of the advocate.

**The Physiological Principles of Hydrology.** By R. G. GORDON, M.D., D.Sc., F.R.C.P., and F. G. THOMSON, M.D., F.R.C.P. Pp. 129. London: J. Cape Ltd. 1930. Price 5s.—Books on hydrology are as a rule rather dull and uninteresting to the general practitioner. The present volume, however, instead of giving details of the chemical constitution of the waters and a list of the Spas which are suited to various diseases, has set forth first the physiology of the skin, circulation, nervous system and metabolism in relation to hydrology, and after discussing the internal and external use of the waters, deals finally with their application to a variety of diseases. We are glad to see that emphasis is laid rather upon the physical than the chemical properties of the waters, and that a warning note is sounded in reference to the treatment of all forms of myocardial disease by Nauheim baths. The historical sketch of the use of hydrology is full of most interesting and amusing matter.

**Shorter Convalescence.** By LIEUT.-COL. J. K. MCCONNEL, D.S.O., M.C. Pp. xi., 130. London: William Heinemann Ltd. 1930. Price 5s.—This little book will be of interest alike to the general practitioner and the specialist, for both classes have at some time under their care cases recovering from acute illness and also those to whom Goldthwait refers as a discredit to the medical profession, *i.e.* the "chronic cases." Col. McConnel's suggestions cannot fail to help to reduce the period of invalidity of the former and prevent many of them from being added to the classes of the chronic. We must all be familiar with cases in whom relief of the acute abdominal condition, for which they were operated on, was associated with the development of chronic backache or flat-foot from lack of attention to postural considerations during their convalescence. The introduction of the simple exercises advocated by Col. McConnel, as a routine for all recumbent patients who have passed the acute stage, would prevent such disasters. The author has made an intensive study of his subject and gives a valuable bibliography, while within the small compass of his book he manages to provide a simple and interesting summary of the most modern work in connection with the maintenance of posture and the re-education of correct posture in subjects who have either failed ever to attain it, or have lost it through illness or strain. The evolution of upright posture in man, its physiology as a series of conditioned reflexes, in which muscles that are antagonists for movement contract synergically, and the influence on the circulation of muscle action and diaphragmatic respiration, are all dealt with briefly but clearly. In his introduction, the author agrees that certain acute conditions "may preclude exercises of even the mildest description," but points out that even soon after a laparotomy, "co-ordinated tightening of the abdominal wall can be made without any pain, especially if this takes place automatically." One must agree with Sir Robert Jones, who in a foreword says "this book deserves a wide circulation."

**The Elements of Medical High Frequency and Diathermy.** By W. C. DOUGLASS, M.R.C.S., D.M.R.E. Pp. viii., 136. 67 illustrations. London: H. K. Lewis & Co. Ltd. 1930. Price 6s.—It is a bold undertaking to explain in a hundred pages the theory and method of producing high frequency and diathermy currents: the more so when the reader is initially supposed to have attained a knowledge of electricity that would hardly take him through the preliminary M.B. examination. While the elementary theory of dynamos and of induced and oscillating currents is clearly expounded, it seems very doubtful whether the intended reader will attain a sound knowledge of high frequency and diathermy from an account necessarily very condensed. The descriptions of diathermy apparatus are little more than the short accounts provided by the various makers for catalogue purposes and the author utters the warning that the practical application of medical electricity must be sought in more extensive works.

**Synopsis of Surgery.** By E. W. HEY GROVES, M.S., M.D., B.Sc., F.R.C.S. Ninth Edition. Pp. viii., 676. Illustrated. Bristol: J. Wright & Sons Ltd. 1930. Price 17s. 6d.—When a book has hastened to a ninth edition, the task of the reviewer is a light one. His only duty is to point out wherein the new edition is an improvement on its forerunners. The sections on surgical shock, spinal anæsthesia, glossitis, tumours of the bladder and testis, and infections of the kidney have been largely rewritten. It is not surprising to find that Professor Hey Groves' own new methods of treating fractures of the neck of the femur are noticed. New articles appear on the injection treatment of varicose veins, tuberculous bladder (we remember calling attention, in reviewing a former edition, to the lack of this) and the Winnett Orr treatment of septic conditions of bone.

**The Mycoses of the Spleen.** By ALEXANDER GEORGE GIBSON, M.D., F.R.C.P. Pp. xii., 169. Illustrated. London: Kegan Paul, Trench & Trubner. 1930. Price 12s. 6d.— In 1912 the author of this book demonstrated mycelial structures in the spleen from a case of Banti's disease. The mycelium was found in the Gandy-Gamna nodules—small fibro-cellular areas with necrotic centres present in the trabeculæ of the spleen in Banti's disease. Since then he has carried out further investigations on spleens from the various forms of splenomegalic disease. The book deals mainly with the author's own work on the subject, but the views and work of French observers are given full prominence, especially those of Professor Nanta who recently claimed to have demonstrated the mycelium of an aspergillus in the Gandy-Gamna nodules in Banti's disease. Following a chapter dealing generally with the splenomegalies there are chapters giving details of the cases investigated, the anatomical and histological appearances of the spleens, the results of splenic culture and the animal tests carried out with the various strains of streptothrix isolated from some of the spleens. The final chapter deals with the mycotic hypothesis of the cause of the splenomegalies and the contribution of French workers. The author is to be congratulated on the very convincing evidence he has produced that splenic anæmia, acholuric jaundice, and Banti's disease have a common etiology and that the causal organism is a streptothrix which invades the spleen. Apart from the other features the book is a veritable storehouse of general information on the splenomegalies, and the physician who has such cases under his care will find in it much that will be of interest and use to him. The illustrations are excellent and there is a good index and a long list of references.

**Nursing and Diseases of Sick Children.** By ALAN MONCRIEFF M.D., B.S., M.R.C.P. Pp. xvi., 550. Illustrated. London: H. K. Lewis & Co. Ltd. 1930. Price 15s.—This book is a product of the great Ormond Street school, and has been written by various members of the staff in collaboration with the Sister Tutor under the editorship of Dr. Moncrieff. Whether such an exhaustive knowledge of the diseases of sick children is necessary to a children's nurse is not for the reviewer to say. But, judging from the questions taken from the State Examinations and included in the book as an appendix, the examiners do not expect such details as the book presents. It is difficult to see why nurses should be worried with such things as the various types of myopathies, the relationship between vaccinia and small-pox, or the pathogenesis and investigation of hydrocephalus. The sections on nursing are excellent. The book is very good as a reference book for children's nurses, but one cannot help feeling that as a text-book it is much too elaborate.

**Chronic Nasal Sinusitis and its Relation to General Medicine.** By P. WATSON-WILLIAMS, M.D. Pp. xvi., 221. 109 illustrations. Bristol: J. Wright & Sons Ltd. 1930. Price 15s.—Dr. Patrick Watson-Williams has enlarged his Semon Memorial Lecture, delivered in 1925, into an admirable book. The subject is one to which his long experience as a general physician before he became an oto-rhino-laryngologist has enabled him to do full justice. His object has been to point out the many toxæmias and secondary infections which may be the outcome of chronic nasal sinusitis. In this he has achieved a very real success because, in addition to a thorough understanding of nasal infections, whether manifest or latent, he is able from his wide knowledge to describe with clarity and precision the symptomatology of the resultant toxæmias and infections, more especially the mental disturbances associated with them. The clinical records which are included in this book furnish brief and striking illustrations of the manifold remote effects of focal sepsis in general and of nasal sepsis in particular. No psychiatrist, no physician and no general practitioner can afford to ignore the information contained in this book. Desultory references may be found in general literature to the systemic infections which depend upon chronic nasal sinusitis, but this is the first book devoted solely to the subject. The technique of nasal endoscopy, of diagnostic exploral suction of the sinuses and the remedial operations are well described and explained. The illustrations are numerous and good. Dr. Watson-Williams deserves hearty congratulations on having written a pioneer work.

**Essays and Addresses: Sociological, Biological and Psychological.** By A SURGEON. Pp. xiii., 277. London: H. K. Lewis & Co. Ltd. 1930. Price 10s. 6d.—These essays vary considerably in interest and importance, but several underlying principles can be traced throughout the series. The importance of the part played by evolution in the most fundamental of human affairs, both in the mental and physical spheres of individual social life, is the keynote. It is forcibly emphasized that all attempts to promote human progress must be based on hereditary constitution and innate capacity. The pressing need of a wider diffusion of biological knowledge in the framing of social legislation and national life is one of their chief notes, and a plea is put forward for a census of the quality as well as the quantity of the nation. An interesting theory is advanced to explain the origin of " use acquirements " through variation and selection occurring among the intra-cellular units of which the animal cell is composed. There is much of value and interest to be found therein, and those interested in these subjects will be well repaid by their perusal.

